# Virtual game-based learning environments to promote self-regulated learning skills in foreign language learners

**DOI:** 10.12688/f1000research.157746.1

**Published:** 2024-12-20

**Authors:** Maira Alejandra Noriega Cortes, Laura Carreño-Bolivar

**Affiliations:** 1 Independent Researcher, Chia, Colombia; 2 Universidad de La Sabana, Chia, Colombia

**Keywords:** Gamification; self-regulated learning- self regulation; Language Learning; virtual learning environment- virtual learning- blended learning environment.

## Abstract

**Background:**

This mixed-method action research study explores the impact of a gamified virtual learning tool on elementary students’ self-regulated learning (SRL) skills in English language acquisition. Despite the acknowledged importance of SRL in educational contexts, limited research exists on its development among young learners. The present study is one of the products of the academic unit project “Key Factors and Essential Elements for a Comprehensive Model of Bilingual Education: A Multidimensional Approach for Diverse Contexts and Areas of Knowledge” sponsored by the School of Education at Universidad de La Sabana, institution where the first author conducted her master’s studies, and the corresponding author is a professor and main researcher of the of the academic unit project.

**Method:**

Six students aged 8-10 participated in the study. Data were collected through semi-structured observations and pre- and post-questionnaires. The tool was designed based on Zimmerman’s SRL model and included tasks structured through gamification elements. For the purpose of quantitative data analysis, descriptive statistics were used; as for qualitative data, thematic analysis was the chosen approach.

**Results:**

The results revealed that guided use of the gamified tool significantly enhanced students’ use of SRL strategies, particularly in goal setting, monitoring progress, and reflecting on learning outcomes. Students demonstrated increased motivation, engagement, and confidence, although explicit instruction was required to maximize the tool’s benefits. The usability of the tool was rated positively, with participants highlighting its effectiveness for language learning and SRL skill development.

**Conclusions:**

The study concludes that gamified virtual environments can effectively promote SRL skills in young language learners by fostering motivation and engagement. However, for optimal outcomes, such tools should be supplemented with explicit instruction in SRL strategies. This research contributes to understanding the integration of gamified tools in elementary education, highlighting their potential to develop foundational skills essential for lifelong learning.

## Introduction

With evolving educational landscapes and changing motivations of new generations, autonomy and awareness of one’s learning process and skills have become essential for successful learning. Being a highly self-regulated learner is an important human resource for students to learn how to learn to manage, regulate, and enjoy their learning ( Fukuda, 2019; Lu et al., 2017; Wang, 2021; Zhou & Li, 2020; Zimmerman, 2000), which is the reason behind a current research phenomenon that attempts to deeply explore self-regulated learning applied to different educative contexts and learning scenarios. Little research has been done on the matter, specifically in Colombia ( Giraldo, 2022; Gómez Sará, 2017; Moreno et al., 2021), leaving a gap in knowledge in terms of how young elementary students develop self-regulated learning skills, how those abilities can be taught inside the language classrooms, and how gamification on digital platforms can be an effective resource in supporting the development of self-regulated learning. Thus, the present study aimed to focus on the interaction between self-regulated learning, language learning, and gamified technologies, and how these variables affect the development of self-regulated learning skills in elementary students. The data collected and the design of a virtual learning environment that can successfully promote self-regulated learning from an early age, became a significant contribution to the educational field as well as to the knowledge field in Colombian research of elementary language learners.

## Theoretical background

### Self-regulated learning

The importance of self-regulated learning and its impact on motivation and academic achievement has been a critical matter for educational research since Albert Bandura and Barry Zimmerman in the late eighties ( Lu et al., 2017). Being one of the main referents of the concept, Zimmerman (2000) conceptualized self-regulation as “self-generated thoughts, feelings, and actions that are planned and cyclically adapted to the attainment of personal goals” (p. 14). After many years of reflection, it was established that self-regulated learning was the result of the combination of a metacognitive component, motivational beliefs, and cognitive and social affective elements ( Lu et al., 2017). Over time, the concept of self-regulated learning has allowed researchers to understand there is a cause-effect relationship between self-regulated learning and academic achievement, as the higher level of self-regulated learning skills learners have, the higher academic results they might obtain ( Fukuda, 2019; Neitzel & Connor, 2018). Hence, self-regulated learning has also been related specifically to language acquisition and how it influences positively certain language skills ( Fukuda, 2019; Yüce, 2019). Research has identified that the more students are immersed in traditional learning environments and traditional learning methods, the lower levels of self-regulated learning are going to be developed ( Li et al., 2022). Because of that, Cerda et al. (2020) described the relevance of a flexible bilingual learning environment that cultivates student choice and supports content meaning-making, self-regulation, and language learning. As a result, self-regulated learning contributes to language learners’ engagement and enhanced performance in the foreign or second language classroom ( Wang, 2021), and under that comprehension, the present research examined the relationship between self-regulated learning and successful language learning strategies as part of elementary school settings.

### Foreign language elementary-level students in Colombia

There is broad research on the matter of self-regulated learning development in higher education levels and adult education contexts ( Uus et al., 2021). Nonetheless, studies made with children in this area support the idea that they are curious and exploratory learners, who from an early age can selectively explore and choose the information they want to receive ( Foushee et al., 2021). For example, Dent and Koenka (2016) established a strong correlation between self-regulated learning and academic achievement from kindergarten to second grade, which then became weaker from third through fifth grade. Research in elementary school settings has described even how students’ age or grade level affected the self-regulated learning orientation strategies students tend to use as they progress to higher grades ( Zhu & Mok, 2018). In Colombia, Elementary schools must ensure students acquire basic knowledge for secondary education while developing cognitive, procedural, and affective skills ( Moreno et al., 2021). Many students, particularly in public schools, have low competencies in essential skills like reading and writing in Spanish, and yet, the Ministry of National Education aims for students to achieve an A1 level in English by the end of elementary school to build on in high school ( Giraldo, 2022; Gómez Sará, 2017). For academic success to happen, children should be able to regulate their emotions and behaviours to adapt to their social and learning environments. This will result in better school readiness and more effective learning processes ( Acar et al., 2022). However, when the goal is to pursue a successful learning process that allows students an integral development as learners, other areas of knowledge and skills should be emphasized ( Gómez Sará, 2017). That is why promoting self-regulated learning development from early stages in education becomes of such relevance for these new generations. Even so, children will not necessarily become sophisticated, self-regulated learners just by maturing along the developmental stages, so learners require formal training and practice from a young age in self-regulated learning strategies to be successfully autonomous learners ( Rameli et al., 2022; Zhu & Mok, 2018). Accordingly, the present research was based on the relevance of knowing how to foster and teach self-regulated learning abilities in language learning as part of the early stages of the educational process, to support the development of higher-order thinking skills and emotional capabilities, according to the developmental stage of elementary students.

### Gamification

Gamification refers to the application of game mechanics, aesthetics, and game thinking in non-gaming settings to engage people, motivate action, promote desired behaviors, learning, and solve problems. It may traditionally include the use of interconnected game components (point and leveling systems, leaderboards, badges, bonuses) and game elements (achievements, competition, rewards, and self-expression) while turning the entire learning process into a gaming event ( Kucher, 2021; Ofosu-Ampong, 2020; Opris et al., 2021). Gamification as a teaching-learning strategy in educational contexts can be seen as an effective way to educate students, so it can increase the level of individual and group motivation, have an emotional and social impact on students, and increase academic achievement and learners’ ability to learn new skills. Gamified applications for second language learning have proven effective in boosting children’s motivation, engagement, and confidence through enjoyable and non-judgmental environments ( Li et al., 2022; Shadiev et al., 2017; Yükseltürk et al., 2018). Dindar et al. (2021) found that gamified cooperation and competition positively impact task effort, learning achievement, and motivation in Chinese university students. Li et al. (2022) observed that younger students (8-10) from Hong Kong benefited from a gamified English learning system, with active users showing improved academic performance and self-regulation skills. Learning sequences should be tailored by following gamification principles, this implies adapting activities and procedures to a gaming thinking system to how a lesson is taught and continuing to develop it based on the feedback from the players, therefore utilizing the elements from game design to improve educational outcomes, activities, and students’ learning experience ( Ofosu-Ampong, 2020). In consequence, for the present research gamification was better understood as described in 2011 by Zichermann & Cunningham, like the process of using game-thinking and game mechanics to engage users and solve problems ( Ofosu-Ampong, 2020). This allowed a comprehensive view of a digital tool designed to foster the development of self-regulated learning abilities through an engaging, cohesive, and structured learning experience, while it contributed to further research in terms of the applicability and benefits for elementary students.

### Digital technologies as learning tools

Digital technologies have been studied as tools that enable personalized and collaborative learning, and that can be used by teachers with different purposes ( Bower, 2017). Research also highlights the importance of designing authentic environments for effective learning, which means they should provide activities with real-world relevance, give opportunities for sharing learning experiences regardless of their level of expertise, and allow reflection and assessment within the tasks ( Shadiev et al., 2017). Moreno et al. (2021) found that multimedia elements in digital tools (image, sound, animation, interactivity) enhance self-learning and linguistic skills (reading, writing, speaking, listening) in Colombian elementary students, offering innovative pedagogical methods. Hung et al. (2018) reviewed empirical evidence on digital game-based language learning, noting that multiplayer online role-playing games are commonly used on personal computers, primarily for English learning among university students. This raises questions about how such platforms can also support the development of self-regulated learning in diverse learner groups. Virtual Learning Environments (VLEs), for example, have been associated with formal learning and with relationships between teachers, students, and schools, as their features and potentialities become an important element within the student’s learning processes ( Alves et al., 2017). VLEs facilitate interaction and interrelation within a continuous communication process, enhance the construction of knowledge and meanings, and promote the formation of habits and attitudes ( Alves et al., 2017). Another notion adopted to describe some technological tools applied to education is the Digital game-based learning (DGBL) environment. This according to the researchers has the potential to offer students situated learning that fosters higher levels of engagement and motivation, provides a creative atmosphere to focus on the task, improves real-world skills, and increases students’ self-esteem and interest in the subjects ( Hafeez, 2021; Kucher, 2021; Taub et al., 2020). Regularly, students encounter challenges when adopting efficient self-regulatory processes such as cognitive, affective, metacognitive, and motivational while learning, DGBLs encourage pupils in their learning processes due to the properties and possibilities of games. These attributes foster effective self-regulated learning and provide students with opportunities to take the initiative in their learning by analyzing, synthesizing, evaluating, and performing higher-order thinking skills ( Liao et al., 2019; Taub et al., 2020). In that sense, it is possible to say the technological tool presented as part of this research is a game-based virtual learning environment, which supports itself with gamification strategies to promote the development of self-regulated learning abilities while favoring the development of communicative skills in a foreign language.

## Method

The present study followed a mixed-method approach that combined qualitative and quantitative data collection methods such as semi-structured observations and two questionnaires, both instruments were fully designed and reviewed by the researchers. The study aimed to understand better the role of gamification on the self-regulated learning process of elementary students learning English as a foreign language.

### Context and participants

Six students from second, third, and fourth Elementary grades between 8 and 10 years old were randomly selected (four girls and two boys) from a private school with over 40 years of experience. Known for its high academic standards and accreditations from global agencies like NEASC and CIS, the school emphasizes forming ethical, responsible global citizens. The sample was small but randomly chosen to ensure credibility and reduce selection bias, aligning with the study’s goal of exploring in depth the shared characteristics of a group exposed to similar educational experiences. There was no specific criterion for the selection of the participants, nor for the type of school, and the decision to limit the sample size was guided by practical constraints, including available resources and the employment relationship of the main researcher with the school, which facilitated convenient access to the participants. Despite these constraints, the study’s design enables a focused exploration of self-regulated language learning experiences among a group of students who, while limited in number, represent a consistent sample in terms of socio-economic background and a similar history of exposure to English as a foreign language since preschool. All participants’ first language was Spanish.

### Type of study

The research utilized a qualitative-dominant mixed-methods design supported by quantitative tools. Mixed methods, as described by Johnson and Onwuegbuzie (2004), combine qualitative and quantitative approaches to comprehensively address research questions. This study employed a convergent design to merge qualitative insights from interviews and observations with quantitative data from surveys and performance tests, providing a holistic view of SRL development.

Mixed methods enrich understanding by integrating diverse perspectives and validating findings through triangulation. The approach enabled an in-depth exploration of SRL processes and the gamified tool’s impact on students’ learning experiences.

### Ethical considerations

For the present research, moral and legal codes were considered regarding the ethical treatment and care of the participants involved with research studies ( Saldaña, 2011). The present study was approved by ACT 23-2024 (October 30. 2024), by the Research & Ethics Sub-Committee from the School of Education at Universidad de La Sabana, the document states that *“The Research and Ethics Sub-Committee of the Faculty of Education approves the project “Virtual Game-Based Learning Environments to Promote Self-Regulated Learning Skills in Foreign Language Learners”, study conducted by the student Maira Noriega Cortés (main author) and Professor Laura Lucía Carreño (corresponding author), the study demonstrated validity, reliability and attached as evidence of the guidelines followed, the signed consent forms by participants, institutional endorsement letter, and instruments”.* Based on the Research and Ethics Sub-Committee guidelines and the globally accepted guidelines for researcher conduct outlined in in UNESCO’s Universal Declaration on Bioethics and Human Rights, for this research it was of special interest to take care of the participants by informing them about the study, sharing the characteristics of their participation, protecting their identities by using pseudonyms, and clarifying that no physical or emotional harm would be caused.

Furthermore, it was explained that the research was being conducted for the benefit of the community, that the information was being guarded and used only for research purposes, and that a responsible action was being performed by being open to all the questions that participants and related members of the community could have. As the main participants were children, only the students whose parents opportunely signed the consent form were included in the research. In addition, the school administration was informed about the project and signed a consent form.

### Methodological design of the virtual-learning environment

**Figure 1.**
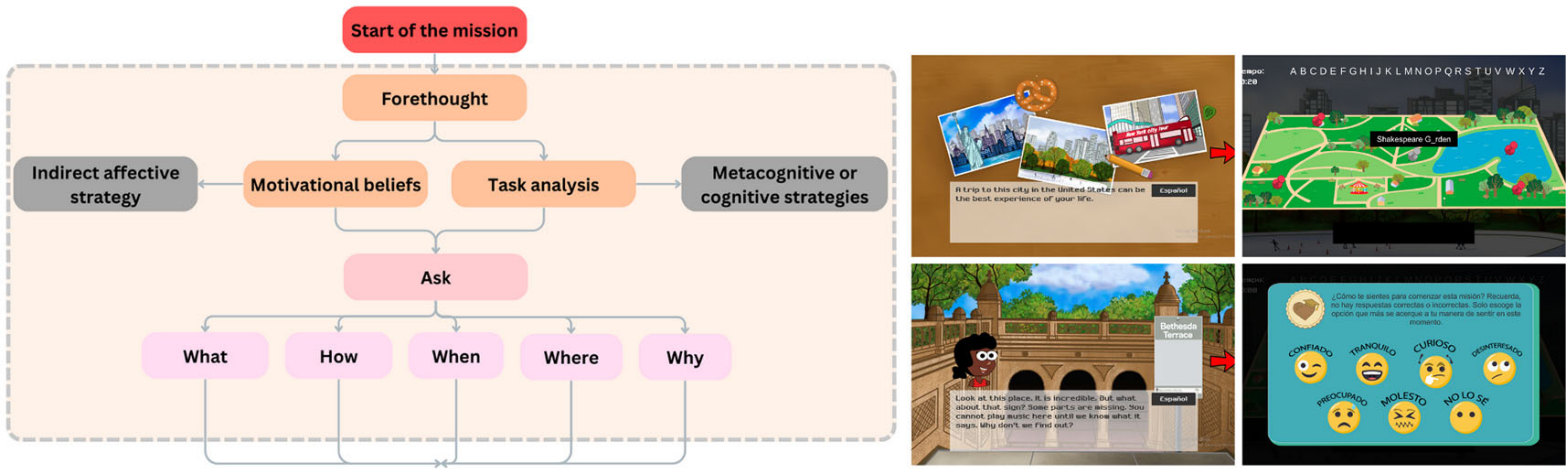
Forethought phase in the self-regulated learning process of the game.

**Figure 2.**
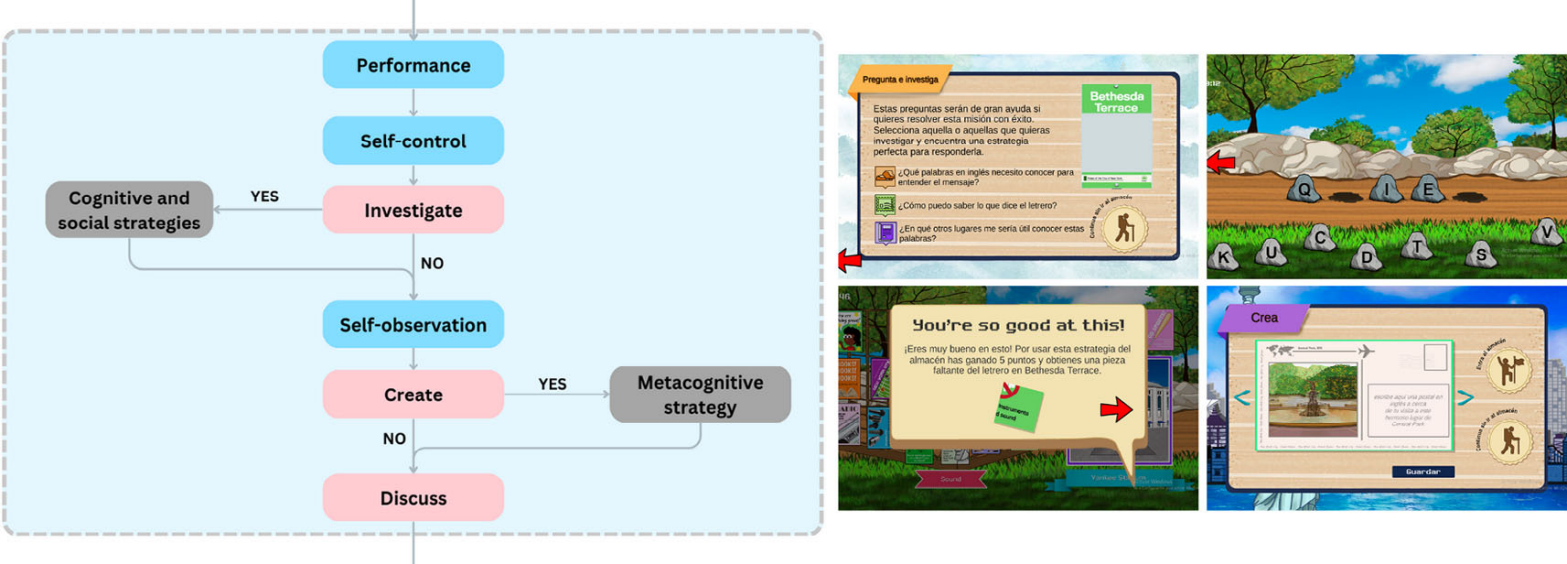
Performance phase in the self-regulated learning process of the game.

**Figure 3.**
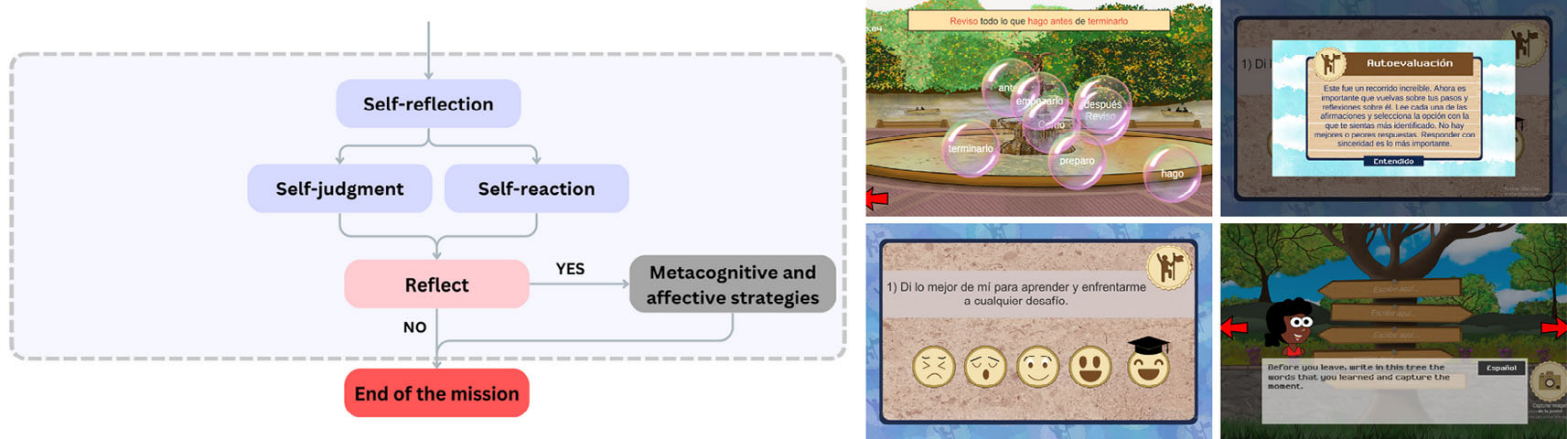
Self-reflection phase in the self-regulated learning process of the game.

The methodological design of the virtual-learning environment, was the skeleton behind the project and focused on three aspects:

1. The structure was based on the self-regulated learning model proposed by Zimmerman (2000) and established three stages to the self-regulated learning process: a forethought (see Figure 1), a performance (see Figure 2) and a self-reflection (see Figure 3).
2. This structure worked because of a system of strategies for learning a language proposed by Oxford (1990), that considered metacognitive, cognitive, affective, and social learning strategies.
3. Finally, it followed a pedagogical route given by the inquiry-based learning model from Bruce and Bishop (2002), so that the communicative tasks took place throughout a cycle of five stages: ask, investigate, create, discuss and reflect. This responded to a specific problem or mission, based on what is established for the levels of A1 and A2 proficiency according to the Common European Framework.

Finally, for the design of the prototype a narrative in which students became travelers, visiting different places where it would be necessary to use English as a foreign language was proposed. The final version prototype of the virtual-learning environment used for this research, was design through the video game engine Unity, and represented one level of the whole game, essentially, one mission that takes place in New York City with 3 essential parts:

1. Introduction (New York Airport): The player is welcome to the city and a role for the mission is assigned. For the 1st mini game, the player must decide the means of transport to get to Central Park:
  • Taxi: Use of resources (Cognitive strategy): the player must find a destination to visit from a New York tourist guide with specific descriptions.
  • Subway/Bike rental: Overviewing and linking (Metacognitive strategy): the player must find a specific area of the park to visit in a Central Park tourist guide with a list of five places of different characteristics.
2. Main activity (Bethesda Terrace in Central Park): The three phases of Self-regulated learning are accomplished considering the inquiry-based learning cycle. It contains five mini games that support the development of the story and two types of evaluations (three self-assessments of motivational beliefs and one self-assessment about the learning process).
  • Anticipation: This stage includes a description of the mission and the first self-assessment of motivational beliefs.
    ○ 2nd mini game: Goal Setting (Metacognitive Strategy): the player must go through a map of the area in Central Park looking for signs with missing letters. The letters are the missing pieces of a sentence that becomes the learning objective for the mission.
  • Development:
    ○ Ask and investigate: the player must select from a series of questions, each of which leads to a different learning strategy:
      ▪ 4th mini game: Contrast (Cognitive strategy): Pairs of stamps must be made based on the words in English and their corresponding translation in Spanish.
      ▪ 5th mini game: Keywords (Cognitive strategy): Different words that are part of the vocabulary of the mission must be formed by organizing a series of rocks with letters located in a section of the park.
      ▪ 6th mini game: Cultural understanding (Social strategy): by selecting the images of a magazine stand that are related to each other, the player unlocks the photo and the name of another place in New York City in which it is necessary to use the presented keywords.
    ○ Create: it begins with the second self-assessment of motivational beliefs.
      ▪ 7th mini game: A sliding puzzle must be solved to assemble a sign located in an area of Central Park.
      ▪ 8th mini game: Monitoring (Metacognitive strategy): the player must pop the bubbles that contain the missing words of the sentence which describes some self-monitoring tasks.
    ○ Discuss: a postcard must be written to describe the most important memories of the player’s visit to Central Park. The postcard can be shared with someone else (partner/teacher) for feedback.
    ○ Self-reflection: it begins with the third self-assessment of motivational beliefs.
      ▪ Self-assessment: The player scores each statement with different emojis according to his/her performance throughout the mission.
3. Closure (Central Park): a specific task related to the mission and the proposed learning objective is performed to conclude the trip to the city.
  ○ The player must go through the map of Central Park to see the signs of different areas and find the most appropriate according to the proposed mission.
  ○ The player also makes a record of the learned words according to the learning objective proposed at the beginning of the mission.

### Data collection instruments and research process

Two primary instruments were designed for data collection: a semi-structured observation format (see Table 2) and questionnaires. These tools were chosen for their ability to generate both qualitative and quantitative data, aligning with the study’s mixed-methods approach. Existing validated instruments, such as the Self-Regulation Assessment Suite ( Heger et al., 2022) and the Intrinsic Motivation Inventory ( Ryan, 1982), informed the design process.

All the participants filled in a pre-questionnaire (see Table 1) with 11 questions on a Likert scale that went from *Never* to *Always,* to identify the self-regulated skills they already had as part of their everyday learning activities.

**Table 1.** Student’s pre-questionnaire. ^1^

PRE-TEST QUESTIONNAIRE					
NAME: _____________________________________ DATE: _________________________________					
ABOUT MY SELF-REGULATED LEARNING	Never 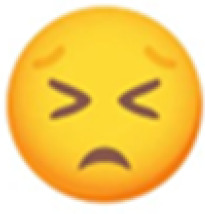	Rarely 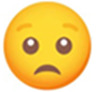	Sometimes 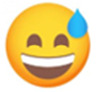	Almost all the time 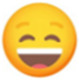	Always 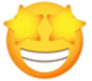
**I recognize that it is important to have learning goals and make sure I fully understand what I need to do before starting an activity.**					
**I recognize that it is important to review my progress while carrying out an activity, to choose the strategies that best help me achieve the goal I set for myself.**					
**I recognize that it is important to evaluate the results of my work at the end of an activity, to identify my strengths and opportunities for improvement.**					
**I set my own learning goals when I am going to start an activity or learn something new.**					
**I reflect on the way I am learning and the results of my activity, to identify my strengths and opportunities for improvement.**					
**I believe I have all the skills I need to be successful in the activity I set out to do.**					
**I can recognize the emotions I am feeling before, during or at the end of an activity.**					
**Depending on the activity and its difficulty, I use different strategies that help me meet the objective and obtain the best possible result.**					
**I can keep my attention on the activity I am doing until I finish it.**					
**I know when to ask or ask an adult for help if I am doing an activity that is difficult for me.**					
**It is easy for me to cooperate with my peers and share the results of my work with them.**					

^1^ Original pre-questionnaire is in Spanish, but it was translated for publishing purposes.

**Table 2.** Observation protocol.

OBSERVATION PROTOCOL			
TESTING PROTOTYPE OF DIGITAL TOOL *BE TO LEARN*			
PARTICIPANT’S NAME:			
DATE OF OBSERVATION:			
TIME OF OBSERVATION:			
TASKS	YES	NO	COMMENTS
SELF-REGULATED LEARNING PERFORMANCE			
The student uses the vocabulary presented to identify the right place to visit during the mission. *Direct cognitive strategy.*			
The student identifies the main purpose of the mission (Complete the missing parts of the park’s sign to find its message). *Indirect metacognitive strategy.*			
The student identifies the learning goal of the mission (Learning 5 words in English). *Indirect metacognitive strategy.*			
The student identifies the meaning of a word in English because of its similarity with a word in Spanish. *Direct cognitive strategy.*			
The student organizes the letters to recognize keywords that are helpful to understand the meaning of the park’s sign. *Direct cognitive strategy.*			
The student establishes the relationship of different images to identify some places in which it is socially and culturally common to use the same vocabulary. *Indirect social strategy.*			
The student recognizes her level of performance during the mission based on the suggested prompts. *Indirect metacognitive strategy.*			
The student asks for support from the teacher during the mission. *Indirect social strategy.*			
The student collaborates with her partners while completing the creation part of the mission (writing a postcard). *Indirect social strategy*			
The student identifies her emotional state at the beginning of the mission, being able to describe how she feels as part of her performance. *Indirect affective strategy.*			
The student identifies her emotional state at the middle of the mission, being able to describe how she feels as part of her performance. *Indirect affective strategy.*			
The student identifies her emotional state at the end of the mission, being able to describe how she feels as part of her performance. *Indirect affective strategy.*			
The student lists the learned words to create a semantic web that allows a greater comprehension of the language. *Direct cognitive strategy.*			
The student discovers that visiting the strategies storage is the only way to get the rewards and collect the missing pieces of the sign to complete the mission.			
The student completes the mission by being able to go through the three stages of the self-regulated learning process (anticipation, performance, and self-reflection).			
**Optional:** The student establishes relationships between different words that are previously known, considering their semantic category or common context of use. *Indirect metacognitive strategy.*			

USABILITY OF THE DIGITAL TOOL	YES	NO	COMMENTS
The student easily understands how to use the digital tool.			
The student finds the instructions clear to follow and accomplish the task.			
The student completes the tasks in a reasonable amount of time considering the design and difficulty of the game.			
The student performs the tasks without making mistakes that do not allow her to continue to the next part of the mission.			
The student finds the strategies to overcome the mistakes that she finds to finish the task.			
The student shows a positive reaction to the use of the game.			

After completing the pre-questionnaire, participants took part in the piloting session of the design virtual-learning environment in which a semi-structure observation was implemented to identify what aspects of self-regulated learning the students could recognize and perform while using the tool. It also served the purpose of collecting information about how the students responded to the use of the tool in terms of its usability (learnability, efficiency, errors and satisfaction). The observation protocol contained 22 observable behaviors and was conducted over three sessions of around 45 minutes, each per couple of students selected per grade.

Once the piloting session was over, students filled in a post-questionnaire (see Table 3) with 17 questions to identify the self-regulated learning skills students used while playing and the game experience in terms of the usability of the digital tool.

**Table 3.** Student’s post-questionnaire. ^1^

POST-QUESTIONNAIRE					
NAME: _______________________________________ DATE: ________________					
	Never 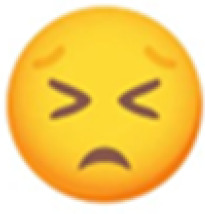	Rarely 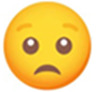	Sometimes 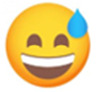	Almost all the time 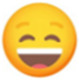	Always 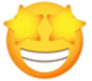
**ABOUT MY SELF-REGULATED LEARNING**					
I was able to recognize the learning goal in the game to ensure I fully understood what I needed to do before starting the mission.					
I was able to review my progress in the game to choose strategies (which appeared in the mini-games) to achieve the goal and complete the mission.					
I was able to evaluate the results of the mission, to identify my strengths and opportunities for improvement.					
I was clear about my learning goal when I started developing the mission as part of the game.					
At the end of the game I was able to reflect on the way I was learning and the results of my mission, identifying my strengths and opportunities for improvement.					
I believe I had all the skills necessary to perform the game activities successfully.					
I was able to recognize the emotions I was feeling before, during, and at the end of all the activities of the game.					
I was able to choose different mini-games with strategies that helped me accomplish the mission objective and obtain the best possible result.					
I was able to maintain my attention throughout the entire mission until I finished it.					
I asked or asked for help from the adult when I encountered parts of the game that were difficult for me.					
It was easy for me to cooperate with my peers and share the results of my mission with them.					
**ABOUT USABILITY OF THE TOOL**					
The game instructions were clear and allowed me to understand what I needed to do to play and move forward.					
I was able to quickly adapt to the game mode to complete the entire mission.					
I was able to complete all of the mission mini games without the game stopping due to malfunctioning.					
I enjoyed the game and everything I was able to do to complete the mission.					
I found it to be a fun tool.					
I found it to be an interesting tool.					

^1^ Original post-questionnaire is in Spanish, but it was translated for publishing purposes.

### Data analysis

Descriptive statistics were used during the first stage in data analysis to understand quantitative data collected through the pre-and post-questionnaires about students’ performance in self-regulated learning skills before and after using the digital tool. The purpose behind following this model was to organize the information and translate it into distribution of frequency, percents, and averages, that provide a summary of relevant numerical information to describe the selected sample, in tables and graphs ( Kaushik & Mathur, 2014). Through this analysis it was possible to understand the relationship between students’ behaviors and their learning experience, by visually representing the frequency of use of a particular behavior, as well as the percentage of increase in the general use of self-regulated learning strategies, before and after using the designed digital tool.

During a second stage a thematic analysis framework was chosen to analyze the qualitative data collected with the observation protocol. It was selected as an analysis procedure since it attempts to find repeated meaning across a data set facilitating the interpretation of a particular phenomenon, after following a rigorous system that includes collecting the data, engaging with it, codify, generate code categories, conceptualize themes, and contextualize and represent the findings ( Braun & Clarke, 2006; Peel, 2020; Xu & Zammit, 2020). Consequently, data from the observation protocol provided information in terms of recurring ideas, behaviors, and strategies exhibited by the students while using the prototype from beginning to end. The analyzed categories are included in the Results section and a comparison with previous research in the Discussion and Conclusion sections.

**Findings**

**Figure 4.**
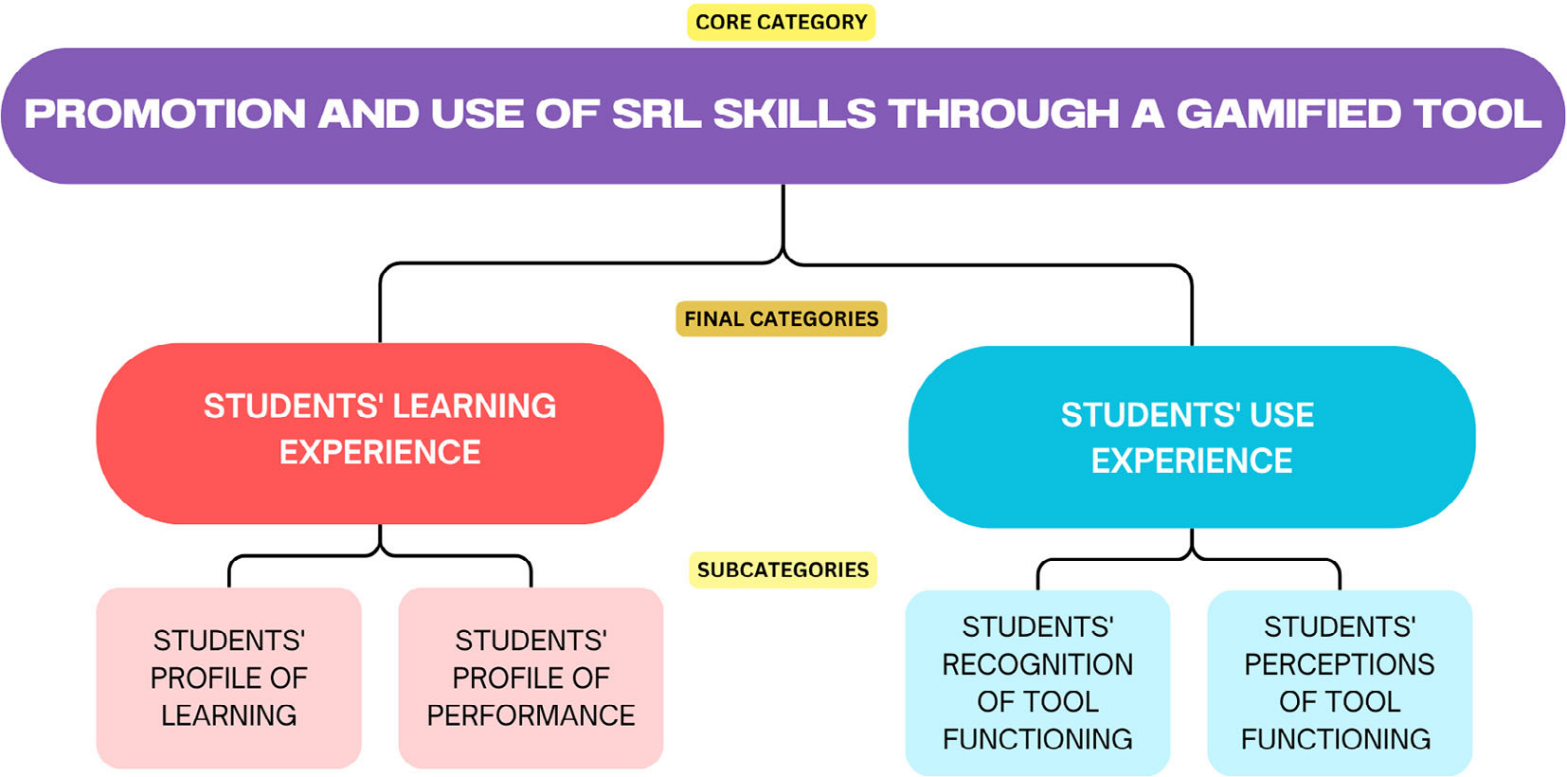
Category mapping.

After careful analysis of the data collected through the observation protocol and the two questionnaires, codes were established for mapping, categorizing and contrasting the qualitative and quantitative data, resulting on a core category and two final categories, each with two subcategories (see Figure 4).

Eventually, this mapping attempted to describe the effects in the development of self-regulated learning skills in elementary students, due to the implementation of a virtual game-based learning tool based on gamification strategies, specifically around the successful learning process of English as a foreign language.

### Students’ learning experience

This final category includes all the data in relation to participants profile of learning based on their self-perception regarding the use of self-regulated learning strategies, and their profile of performance based on the strategies they implemented while using the gamified tool.

In terms of their self-perception of the use of self-regulated learning strategies daily for learning, they frequently question or ask for support when facing challenges during a task, which are common and basic social strategies they use to understand and indicate involvement inside the classroom. In contrast, their less frequent practices include establishing personal goals of learning before starting an activity and monitoring their own progress to modify the implementation of a particular strategy. In general, they perceived themselves performing between three (27%) and eight (73%) of the 11 behaviors in the pre-questionnaire almost all the time, which describe a profile of learning with some development of self-regulated learning as part of the school environment and learning exposure (see Table 4).

**Table 4.** Percentage of behaviors according to frequency of daily occurrence.

Student	Never	Rarely	Sometimes	Almost all the time	Always
S1	0%	9%	27%	36%	27%
S2	0%	0%	36%	27%	36%
S3	0%	9%	36%	36%	18%
S4	0%	0%	9%	36%	55%
S5	0%	0%	9%	36%	55%
S6	0%	0%	27%	73%	0%

*Note.* The table presents the frequency with which the six interviewed students perceive themselves using the 11 behaviors related to self-regulated learning.

On the other hand, their profile of performance was analyzed by paying attention to students’ behaviors while playing during the piloting session (see Table 5). In correspondence to students’ perceptions, while using the tool students were aware of their emotional states, sought adult support when facing challenges, and identified their strengths and needs based on the difficulty of every game. Each student had a unique profile that influenced their learning and use of strategies, which supported the observable evidence that a minimal development of these self-regulated learning abilities is required if the tool is to be used independently by students. Part of learning technologies effectiveness depends on students’ prior experiences, skills, personalities, and circumstances, inevitably students with attentional or regulatory difficulties often would need significant teacher support during group exercises to follow instructions, stay focused, and identify strategies for success. This highlights the importance of pairing such tools with explicit instruction in self-regulated learning and learning strategies to maximize their benefits inside and outside the classroom.

**Table 5.** Percentage of behaviors according to frequency of occurrence during piloting.

Student	Never	Rarely	Sometimes	Almost all the time	Always
S1	9%	18%	9%	9%	55%
S2	0%	18%	18%	18%	45%
S3	0%	0%	0%	45%	55%
S4	0%	0%	0%	36%	64%
S5	0%	0%	18%	9%	73%
S6	0%	0%	9%	91%	0%

*Note.* The table presents the frequency on which the six interviewed students perceive themselves using the 11 behaviors related to self-regulated learning while using the gamified tool.

The post-questionnaire also provided information about students’ profile of performance in which participants perceived themselves confident and with the necessary skills to perform all the proposed tasks, having different options of strategies to achieve the learning objectives of the mission. In contrast, they felt they did not consciously monitor their progress during the game, and some struggled to maintain attention due to the interaction and game dynamics with their partner, which also evidence consistency with the observable behaviors during the piloting, and their answers on the pre-questionnaire.

### Students’ use experience

This final category includes all the data in relation to participants recognition and perceptions of tool functioning considering usability aspects such as learnability, efficiency, errors, and satisfaction. Overall, all students understood the tool, completed tasks within the expected time, and reacted positively to its narrative and appearance. Students first learned the game’s functional aspects, like dragging or clicking with the mouse, and although they struggled while discovering technical features, once they grasped the general mechanics they navigated the tool easily. The results from the post questionnaire in relation to the usability of the tool, reflected students’ enjoyment of the tool considering it fun, creative, exciting and interesting, which correlates with their positive verbal and physical responses observed while using the tool. Some students referred occasional difficulties understanding instructions, which also comes as a result from those participants who out of impulsiveness needed more time and peer/teacher support to adjust to the game mode and dynamics.

At the end of the piloting sessions, it was a recurring idea for the participants the ways in which the tool could be a useful resource in English class to learn vocabulary, language use, and even develop a grater comprehension about cultural aspects. Nonetheless, none perceived the tool as a resource to perform and improve their self-regulated learning process, nor identify these strategies as learning assets to improve their ways of learning in other contexts.

### Promotion and use of self-regulated learning skills through a gamified tool

**Figure 5.**
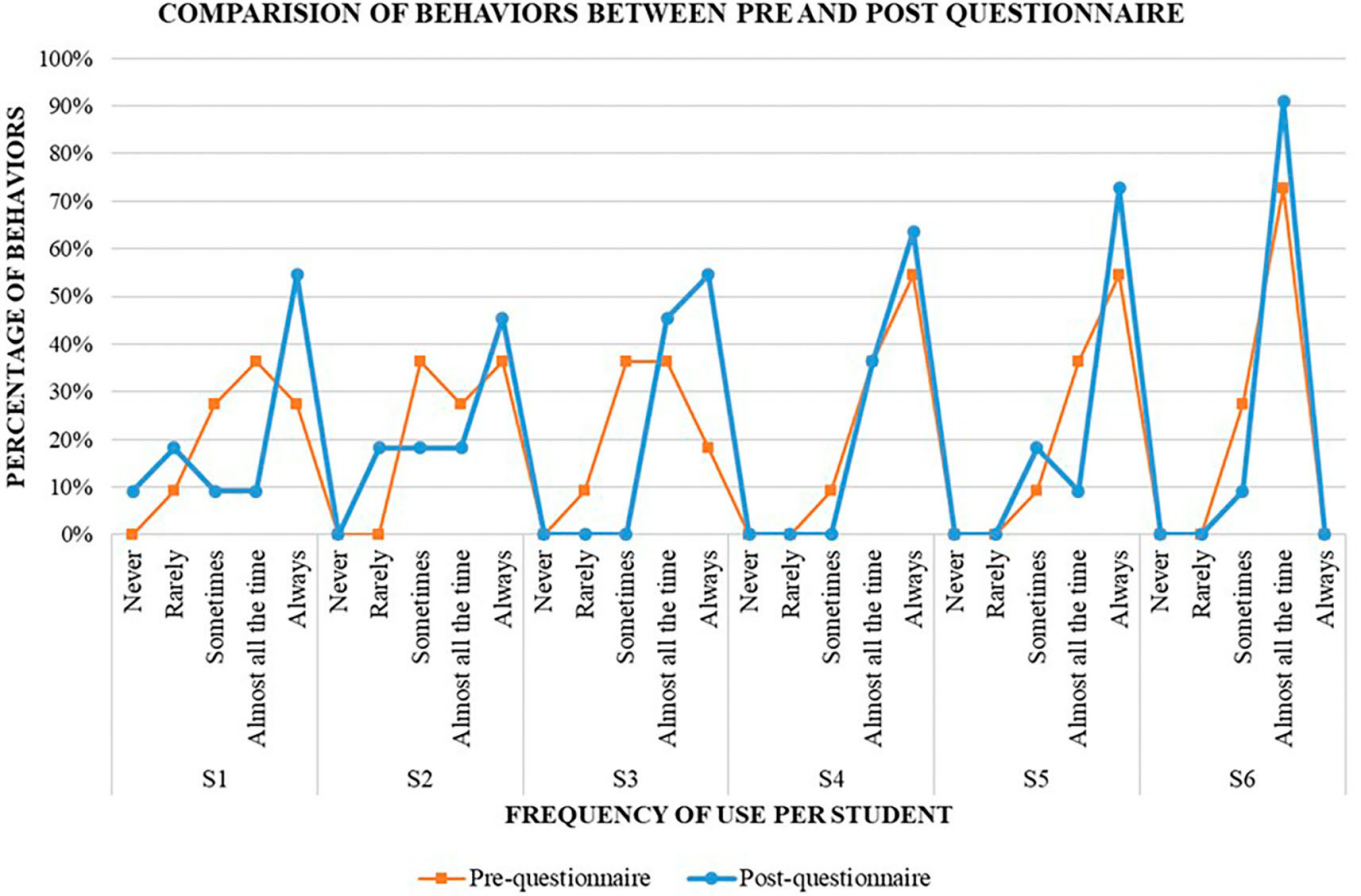
Comparison of students' pre-questionnaire and post-questionnaire results on the frequency of employment of SRL behaviors before and while using the gamified tool.

The whole process of testing the tool included the three stages of self-regulated learning and allowed students to use a set of eleven language learning strategies. Following the implementation of the tool, students’ self-assessments demonstrated a more lucid comprehension of the pedagogical objectives underlying the assigned task. Additionally, there was a marked increase in their self-efficacy and confidence in their abilities to attain these objectives. The use of diverse learning strategies became more prevalent, facilitating their achievement of desired outcomes. Furthermore, the tool provided an expanded scope for collaborative engagement with peers (see Figure 5), thereby enhancing the overall learning experience.

Likewise, when stablishing a comparison between the percentages of use from the pre-questionnaire and the post-questionnaire, all the students had an increase of use from between 9% and 36%, in those self-regulated learning behaviors that occurred always as part of their learning process. This implies that employing a gamified tool with a coherent, systematically and attractive designed framework, which facilitates students’ guided exploration of various self-regulated learning strategies and influenced students’ engagement, emotions and behaviors, can significantly enhance their self-regulated learning performance.

## Discussion

The findings of this study indicated that a structured tool guiding the learning process and incorporating gamification significantly enhances students’ purposeful recognition and implementation of self-regulated learning strategies. Employment of this tool enabled students to approach tasks with a clear objective, thereby reinforcing their confidence and expanding their repertoire of learning strategies within diverse game-based scenarios. Collaborative efforts were also prominently integrated into students’ performance, enriching their application of social strategies to seek support, and receiving comprehensive feedback on language use. As such, it is of higher value in educational search to understand which factors can influence the fostering of self-regulation towards greater motivation and academic achievement.

First, it is important that students can approach self-regulated learning consciously and explicitly by utilizing resources that provide a safe, engaging, and scaffolded environment. Previous research has addressed that instruction interventions in self-regulated learning help learners intentionally and goal-directedly enhance their language learning. Metacognitive, social, affective and cognitive interventions should include explicit instruction that emphasizes consciousness-raising regarding the whole learning process through modeling of strategies for goal setting and planning, guiding practice within a safe environment to internalize strategies in a structured manner, promoting self-monitoring, and providing feedback and evaluation to identify strengths and weaknesses ( Azatova, 2021; Bai & Guo, 2018; Chen, 2022).

On the other hand, tool design and gamified elements should be considered given that, as evidenced in this research, a score based on points that students obtained by using learning strategies, penalization for not accessing the storage of strategies to complete mini games, and offering students a total score with visual rewards at the end of the mission, were game elements that increase participants’ motivation, self-efficacy and enjoyment in the learning process. This is also supported by previous research that describe how gamified elements like narrative and storytelling, progress tracking bars, levels, personal profiles, experience points, badges, rewards, bonuses, penalties, challenges and skill trees, are elements that relate to autonomy and competency, provide feedback during the learning experience, and enhance students’ performance and participation by making the learning more engaging and interactive, while supporting improved academic outcomes for a diverse student population ( Chapman et al., 2023; Chapman & Rich, 2018; Nabizadeh et al., 2021).

In the end, this research investigated the impact of a virtual game-based learning tool employing gamification strategies on elementary students’ development of self-regulated learning skills, and results from piloting sessions showed positive effects on participants’ learning outcomes and motivation. This study contributed to understand the importance of early instruction in self-regulated learning processes for elementary students, aligned with the findings on the emergence of self-reflection and metacognitive skills in children aged 8 to 10 and younger ( Goetz et al., 2013), and highlighting the impact that early instruction could have on students’ foundational stage for their education. Additionally, the creation of the virtual learning environment was a valuable contribution based on the analysis of a wide range of theoretical and practical information, that exposed how self-regulation can be promoted while students completed gamified tasks and learned another language. The tool’s instructional design, narrative, visuals, and game elements significantly enhanced students’ engagement and motivation, pointing out the value of gamified learning experiences in fostering self-regulated learning.

Furthermore, the research underscores the significance of preparing students for success in a knowledge-driven society by developing 21st-century skills, including self-direction, collaboration, critical thinking, emotional intelligence, initiative, and global awareness. Integrating tools like the one designed for this research into educational settings to equip students with essential skills beyond language acquisition or standardized testing, is key to cultivate students’ autonomy, self-awareness, and adaptive learning strategies from an early age to meet future challenges effectively. As such, this can be a replicable research experience to continue raising awareness about the broader implications of implementing such tools across diverse educational contexts, and prompting considerations about how students from various sociocultural backgrounds can develop self-regulated learning skills.

## Conclusion

Self-regulated learning is a core competency to prepare learners for modern society’s challenges at large, making it crucial to provide meaningful opportunities for students to understand, learn, practice and refine these skills and routines in any knowledge environment they experience ( Boekaerts & Corno, 2005; Goetz et al., 2013; Li et al., 2022). The study concluded that the designed digital tool effectively facilitated students’ exploration of self-regulated learning strategies through the processes of forethought, performance, and self-reflection in a language learning task. The gamified elements of the tool promoted engagement and positive emotional responses to learning challenges. However, the tool alone is insufficient for a deep understanding of self-regulated learning, highlighting the need for explicit instruction and practice in language learning strategies alongside the tool’s use. Moreover, there is a need to integrate more theory and research into language teaching pedagogies to establish best practices for introducing self-regulated learning to elementary students. The research demonstrated that gamified virtual environments significantly enhance student engagement in language learning tasks, making learning enjoyable and interactive, turning this approach into a direct strategy to foster self-regulated learning in young learners, promoting early intervention benefits that extend beyond language learning to the acquisition of lifelong skills.

A few limitations must be considered when interpreting the results of this study. Firstly, while the gamified tool employed in the research incorporated Zimmerman’s three stages of self-regulated learning, it only examined the effects of 11 selected learning strategies, potentially overlooking the influence of other strategies on outcomes. Additionally, due to constraints in prototype development, the study could not explore multiple levels of the game or extend testing over a longer period to explore long-term impact and applicability to a wider student population, limiting insights into the tool’s efficacy with a broader array of strategies and sustained use. Consequently, the study’s findings are restricted by the short duration of exposure to the tool and the small participant sample, limiting the ability to generalize qualitative findings. These limitations underscore the need for further research with larger and more diverse samples to better understand the potential benefits and challenges of integrating gamified learning tools into elementary education ( Alshammari, 2020). Small datasets like the one in this study can contribute to future research by being included in meta-analyses, and the findings can eventually be combined with similar research, increasing the overall value of the collected information.

Further research avenues in the field of promoting self-regulated learning skills among elementary students can enhance both teaching practices and educational outcomes. Firstly, investigating teachers’ perceptions of gamified tools and their readiness to model and teach self-regulated learning skills to determine the best ways to keep promoting effective development of these skills in elementary students. Additionally, assessing the learning strategies students already use as part of their learning context and their effectiveness when using virtual tools more autonomously is essential. This analysis can provide personalized learning approaches tailored to individual students’ needs and preferences, facilitating the development of self-regulated learning skills through a scaffolded pedagogical approach. Finally, documenting the effects of interventions in language classrooms, beyond language acquisition, is imperative to understand how explicitly teach self-regulated learning strategies, foster a more self-regulated learning environment in the language classroom, and complement digital tool usage, recognizing the effectiveness of these interventions in younger learners, including preschoolers and elementary students. These research directions promise to enrich the understanding and implementation of self-regulated learning interventions in elementary education, paving the way for more effective teaching practices and improved language learning outcomes.

## Ethics and consent

For the present research, moral and legal codes were considered regarding the ethical treatment and care of the participants involved with research studies ( Saldaña, 2011). The present study was approved by ACT 23-2024 (October 30. 2024), by the Research & Ethics Sub-Committee from the School of Education at Universidad de La Sabana, the document states that *“The Research and Ethics Sub-Committee of the Faculty of Education approves the project “Virtual Game-Based Learning Environments to Promote Self-Regulated Learning Skills in Foreign Language Learners”, study conducted by the student Maira Noriega Cortés (main author) and Professor Laura Lucía Carreño (corresponding author), the study demonstrated validity, reliability and attached as evidence of the guidelines followed, the signed consent forms by participants, institutional endorsement letter, and instruments”.* Based on the Research and Ethics Sub-Committee guidelines and the globally accepted guidelines for researcher conduct outlined in in UNESCO’s Universal Declaration on Bioethics and Human Rights, for this research it was of special interest to take care of the participants by informing them about the study, sharing the characteristics of their participation, protecting their identities by using pseudonyms, and clarifying that no physical or emotional harm would be caused.

Furthermore, it was explained that the research was being conducted for the benefit of the community, that the information was being guarded and used only for research purposes, and that a responsible action was being performed by being open to all the questions that participants and related members of the community could have. As the main participants were children, only the students whose parents opportunely signed the consent form were included in the research. In addition, the school administration was informed about the project and signed a consent form.

## Data Availability

Open Science Framework (OSF) repository: Virtual game-based learning environments to promote self-regulated learning skills in foreign language learners, DOI: 10.17605/OSF.IO/MQT3K. ( Noriega-Cortés & Carreño-Bolívar, 2024). This project contains the following underlying data:
• Data Matrix_NORIEGA-CARREÑO 2024.xlsx • Data Matrix_NORIEGA-CARREÑO 2024 ENGLISH.xlsx Data are available under the terms of the CC0 1.0 Universal
