# Supplementary material for: Virtual game-based learning environments to promote self-regulated learning skills in foreign language learners
Source: F1000Res. 2024 Dec 20;13:1541. [Version 1] doi: 10.12688/f1000research.157746.1 (PMC11927079; doi:10.12688/f1000research.157746.1)
Supplement: Peer review history

# Reviewer report: Reviewer response for version 1

**DOI:** 10.5256/f1000research.173242.r355104

**Published:** 2025-02-10

**Authors:** BAMBANG WIDI PRATOLO

**Affiliations:** 1 Universitas Ahmad Dahlan, Yogyakarta, Special Region of Yogyakarta, Indonesia

The study addresses an important gap in research by exploring the development of self-regulated learning (SRL) skills in young foreign language learners through gamified virtual environments. Although SRL has been widely studied in higher education and adult learning, its application to elementary-level language learners remains underexplored. This makes the study a valuable contribution to both educational technology and language acquisition research.

 The use of a mixed-method action research approach strengthens the study’s reliability. By incorporating both quantitative (descriptive statistics) and qualitative (thematic analysis) data, the research provides a more comprehensive understanding of the impact of gamified learning tools on SRL. The study is grounded in Zimmerman’s SRL model **,** which is widely recognized in educational psychology. This theoretical foundation enhances the study’s credibility, ensuring that the research is not only exploratory but also conceptually sound.

 However, the sample size is not sufficient, especially for quantitative research. It is sufficient for qualitative but not for quantitative analysis. If the author can distribute the questionnaires to all the participants in both groups (I believe there should be more participants), it will be much better. A larger sample size would strengthen the validity of the conclusions and provide more robust evidence of the tool’s effectiveness. Besides, The authors need to explain more clearly how they develop the instruments and how they validated them. Also, they need to explain in brief their claim on triangulation in this current study.

 The findings suggest that gamification can enhance SRL skills, particularly in goal setting, monitoring progress, and reflection. The study’s emphasis on the need for explicit instruction alongside gamification offers practical insights for educators designing digital learning environments.

Is the work clearly and accurately presented and does it cite the current literature?

Partly

If applicable, is the statistical analysis and its interpretation appropriate?

Yes

Are all the source data underlying the results available to ensure full reproducibility?

Yes

Is the study design appropriate and is the work technically sound?

Yes

Are the conclusions drawn adequately supported by the results?

Yes

Are sufficient details of methods and analysis provided to allow replication by others?

Partly

Reviewer Expertise:

ELT, TESOL, TEFL

I confirm that I have read this submission and believe that I have an appropriate level of expertise to confirm that it is of an acceptable scientific standard, however I have significant reservations, as outlined above.

# Reviewer report: Reviewer response for version 1

**DOI:** 10.5256/f1000research.173242.r355106

**Published:** 2025-01-27

**Authors:** Mohammad Taufiq Abdul Ghani

**Affiliations:** 1 Universiti Pendidikan Sultan Idris, PERAK, Malaysia

1. **Expand Literature Review:**
  Include recent studies on digital game-based learning and SRL to strengthen the theoretical foundation and provide a more comprehensive background.
2. **Clarify Instructions in the Tool:**
  Some students reported difficulties understanding instructions. Consider refining the clarity and conciseness of the instructions in future iterations of the tool.
3. **Address Generalizability:**
  Discuss the generalizability of the findings given the small, homogenous sample size. Future research could explore the tool's efficacy in diverse educational contexts.
4. **Explicit SRL Instruction:**
  Highlight the need for explicit instruction in SRL strategies alongside the use of the tool, as evidenced by students' inconsistent self-monitoring and reflection behaviors.

Is the work clearly and accurately presented and does it cite the current literature?

Yes

If applicable, is the statistical analysis and its interpretation appropriate?

Partly

Are all the source data underlying the results available to ensure full reproducibility?

Yes

Is the study design appropriate and is the work technically sound?

Yes

Are the conclusions drawn adequately supported by the results?

Yes

Are sufficient details of methods and analysis provided to allow replication by others?

Yes

Reviewer Expertise:

educational technologoly, teaching foreign langauge, education, instructional design, instructional technology,

I confirm that I have read this submission and believe that I have an appropriate level of expertise to confirm that it is of an acceptable scientific standard.

# Reviewer report: Reviewer response for version 1

**DOI:** 10.5256/f1000research.173242.r355097

**Published:** 2025-01-14

**Authors:** Mahjabin Chowdhury

**Affiliations:** 1 Texas A&M University, College Station, Texas, USA

Overall, this is a well-conducted study that makes a valuable contribution to understanding how gamified environments can support SRL development in young language learners. With some minor revisions to strengthen methodological clarity, it will be even more valuable to researchers and practitioners.

 Recommendations for improvement:

1. Methods section should include:

- More details about observation protocols and coding
- Information about instrument validation
- Clearer explanation of data analysis procedures

1. Results section would benefit from:

- More direct quotes from qualitative data
- Additional details about individual student progress
- Clearer presentation of pre/post comparisons

1. Discussion should:

- More explicitly address study limitations
- Provide more specific recommendations for practitioners
- Include deeper analysis of unexpected findings

Is the work clearly and accurately presented and does it cite the current literature?

Yes

If applicable, is the statistical analysis and its interpretation appropriate?

Yes

Are all the source data underlying the results available to ensure full reproducibility?

Yes

Is the study design appropriate and is the work technically sound?

Yes

Are the conclusions drawn adequately supported by the results?

Yes

Are sufficient details of methods and analysis provided to allow replication by others?

Yes

Reviewer Expertise:

Game-based learning, Scholarship of teaching and learning, translanguaging, Co-design, learning experience design

I confirm that I have read this submission and believe that I have an appropriate level of expertise to confirm that it is of an acceptable scientific standard, however I have significant reservations, as outlined above.
